# Biosurfactant-mediated biodegradation of straight and methyl-branched alkanes by *Pseudomonas aeruginosa *ATCC 55925

**DOI:** 10.1186/2191-0855-1-9

**Published:** 2011-05-27

**Authors:** Carlos A Rocha, Ana M Pedregosa, Fernando Laborda

**Affiliations:** 1 Laboratory of Oil and Air Microbiology, Cell Biology Department, Simón Bolívar University, Valle de Sartenejas, Apto. 89.000, Caracas 1080-A, Venezuela; 2 Laboratory of Microbiology I, Microbiology and Parasitology Department, Alcalá University, Carretera Madrid-Barcelona, Km 33, 28871 Alcalá de Henares, Madrid, Spain

**Keywords:** Biodegradation patterns, alkanes biodegradation, biosurfactant, *P. aeruginosa*, cell adaptations

## Abstract

Accidental oil spills and waste disposal are important sources for environmental pollution. We investigated the biodegradation of alkanes by *Pseudomonas aeruginosa *ATCC 55925 in relation to a rhamnolipid surfactant produced by the same bacterial strain. Results showed that the linear C11-C21 compounds in a heating oil sample degraded from 6% to 100%, whereas the iso-alkanes tended to be recalcitrant unless they were exposed to the biosurfactant; under such condition total biodegradation was achieved. Only the biodegradation of the commercial C12-C19 alkanes could be demonstrated, ranging from 23% to 100%, depending on the experimental conditions. Pristane (a C19 branched alkane) only biodegraded when present alone with the biosurfactant and when included in an artificial mixture even without the biosurfactant. In all cases the biosurfactant significantly enhanced biodegradation. The electron scanning microscopy showed that cells depicted several adaptations to growth on hydrocarbons, such as biopolymeric spheres with embedded cells distributed over different layers on the spherical surfaces and cells linked to each other by extracellular appendages. Electron transmission microscopy revealed transparent inclusions, which were associated with hydrocarbon based-culture cells. These patterns of hydrocarbon biodegradation and cell adaptations depended on the substrate bioavailability, type and length of hydrocarbon.

## Introduction

Leaking from oil wells, tanks, pipes and transportation vehicles together with the inadequate waste disposal from the oil industry at large (oil exploration and recovery) have become important sources of environmental contamination (Leahy and Colwell 1990). Alkanes, particularly n-alkanes, are important components of crude oils and its derivatives, such as heating oil, jet fuel, gasoline and kerosene (Marin et al. 1995; Berekaa and Steinbüchel 2000). In nature, some microorganisms oxidize aerobically (Berekaa and Steinbüchel 2000; Solano-Serena et al.2000; Dutta and Harayama 2001) and anaerobically (Chayabutra and Ju 2000; Kniemeyer et al. 2003), co-metabolize (Whyte et al. 1997; Garnier et al. 2000) and detoxify most of the C4-C20 compounds from linear, branched and cyclic alkanes (Scott and Finnerty 1976; Leahy and Colwell 1990), including low-carbon hydrocarbons, which may affect cell membrane integrity (Marin et al. 1995). Particularly, alkanes that are metabolized via oxidation are used as a carbon source for cell growth. Generally, oxidation of alkanes occurs by terminal C-H oxidation followed by β-oxidation. Alternatively, bacteria use α, ω, and Finnerty oxidations as well as β-alkyl group removal by β-descarboxymethylation (Schaeffer et al. 1979). The fate of alkanes during the biodegradation process can be used as a practical tool for assessing bioremediation of oil-polluted sites, which involves some biological-based engineering techniques to improve the microorganisms' ability to biotransform the contaminant to a less or non-toxic state (mineralization), resulting in a more economic and environmentally friendly approach.

Besides the effects of environmental conditions on oil biodegradation, other factors intrinsic to oil, such as oil solubility, partition coefficient, dissolution rate, viscosity and physical state become rate-limiting in the cell-oil uptake and biodegradation by the cell. Consequently, only a small fraction of hydrocarbons will be present in the bulk water phase ready for oxidation, co-metabolism or detoxification (Zhang and Miller 1992), most of it being concentrated in the oil-water interface. In response to this, cell adaptations to growth on oily substrates are also depicted in nature. Particularly, biosurfactant production can occur, which would enhance oil dispersion into the aqueous phase and retard volatilization of low carbon atom-hydrocarbons (below C7), favoring biodegradation (Kretschmer et al. 1982; Neu 1996; Bruheim et al. 1999; Rocha et al. 1999; Rocha et al. 2000). However, this type of amphipathic molecules can also render inhibitory and neutral effects (Bruheim et al. 1999). Despite of that, surfactants, especially rhamnolipidic biosurfactants, have been reported to enhance the biodegradation of crude oil (Rocha and Infante 1997) and many other oil derivatives (Zhang and Miller 1992; Zhang and Miller 1994; Zhang and Miller 1995; Al-tahhan et al. 2000). This type of tensio-active glycolipids are produced by some strains of *Pseudomonas aeruginosa*, which also depict the ability to undertake the oxidation of a wide variety of oil components, including alkanes. In addition to biosurfactant production, cell-to-cell and cell-to-substrate interactions play an important role on alkane biodegradation. In relation to this, hydrophobic compounds can alter cell membranes (Heipieper and Bont 1994; Whyte et al. 1999), including cell surface hydrophobicity, which enhances adhesion of cells to hydrocarbons in the water-hydrocarbon interface (Scott and Finnerty 1976; Rosenberg 1991; Baldi et al. 1999) and transportation through the cell membrane. In response to all these factors, oil-biodegrading bacteria have shown different patterns of alkane oxidation.

In this study we investigated the patterns and kinetics of alkane degradation by a biosurfactant-producing *Pseudomonas aeruginosa *(ATCC 55925) grown on natural heating oil (mainly of 11-21 carbon atoms) and commercial n-alkanes (from C7 to C19 carbon atoms) in relation to biosurfactant. Cell growth and CO_2 _production are commonly used as indirect indicators of hydrocarbon biodegradation; however, these techniques do not demonstrate the real changes that hydrocarbons suffer when they are used as carbon sources for cell growth, such as the degree of hydrocarbon depletion, the patterns of hydrocarbon biodegradation and other cell-hydrocarbon and hydrocarbon-hydrocarbon interactions. In this study we followed directly the hydrocarbon biodegradation by analyzing the substrate through the Gas chromatography technique. Also, some structural and morphological cell strategies for the uptake of hydrocarbons were elucidated by electron microcopy.

## Materials and methods

### Microorganism

*Pseudomonas aeruginosa *ATCC 55925 is a biosurfactant-producing microorganism able to biodegrade a wide range of oily substrates (Rocha and Infante 1997). This strain was isolated from a soil sample continuously exposed to gasoline residues.

### Materials

The extracting solvent n-hexane was purchased from Riedel-de Haën. Pure C7-C22 n-alkanes and C19 branched alkane 2, 6, 10, 14-tetramethylpentadecane (pristane) were obtained from Sigma. Other chemicals were acquired from Riedel-de Haën, Aldrich, Merck, Sigma or Difco at the highest available purity. Heating oil ranging from C11 to C21 carbon atoms was obtained from Repsol oil company.

### Media and culture conditions

*P. aeruginosa *ATCC 55925 was grown in 250 ml-cotton-plugged conical flasks containing 50 ml of a mineral medium described by Bushnell and Hass (Bushell and Haas 1941) and 1% (v/v) inoculum. These cell cultures (biotic systems) were supplemented with one of the following carbon sources: (a) heating oil without additives mainly comprised of hydrocarbons with 11 to 21 carbon atoms (0.5% v/v); (b) C7-C18 n-alkanes and C19 branched alkane (2,6,10,14-tetramethylpentadecane) supplemented individually (0.5% v/v each hydrocarbon) and (c) as a mixture containing a total of 0.5% v/v of all hydrocarbons (0.04% v/v each hydrocarbon). In addition, some cell cultures were further supplemented with biosurfactant 1X its critical micellar concentration (1.5% v/v). Cultures were incubated at 28°C on a rotator shaker at 200 rpm for 20 days. Samples were withdrawn after 0, 5, 10, 15 and 20 days of incubation for hydrocarbon extraction and gas chromatography (GC) analyses. *P. aeruginosa *ATCC 55925 was stored at 4°C on nutrient agar plates and transferred each 15 days. Inocula of *P. aeruginosa *ATCC 55925 were standardized by adjusting the absorbance A_620 _at 0.5. Cell-free controls (abiotic systems) were incubated under the same conditions stated above with and without biosurfactant.

### Production of biosurfactant

Rhamnolipid biosurfactant produced by *P. aeruginosa *ATCC 5592 was obtained as described before (24). Partial purification was undertaken as follows: 1 L of culture was sterilized at 15 *psi *for 15 min. Cell supernatants obtained after centrifugation at 9,000 g, for 20 min at 4°C were acidified with HCL 2N to pH 3.0. Ramnolipids were extracted with diethyl ether under continuous agitation for 12 h. The solvent phase was evaporated in vacuum and the residual rhamnolipid was suspended in deionized water to a final concentration of 0.1 mg/ml.

### Analytical Methods

Quantification of hydrocarbons was determined as follows: After incubation and just previous to extraction with n-hexane, 100 μl of pure n-decane was added to the culture broth as an internal standard against which all hydrocarbon depletion was corrected. n-decane was chosen as it eluted before heating oil and pure hydrocarbons in the gas chromatography profile. Hydrocarbons were then extracted with three successive treatments of 5 ml n-hexane. The organic phases were combined, the volume was adjusted to 25 ml using n-hexane and the extracts were analyzed by GC.

For gas chromatographic analysis (GC) one μl of sample was injected in a gas chromatograph (Hewlet-Pakard model-5890 series II) equipped with a flame ionization detector and an ultra 1 (dimethylpolysiloxane) capillary column (25 m long × 0.2 mm diameter). The oven temperature was increased from 80°C to 280°C at a rate of 8°C.min-^1^. The injector and detector temperature were set at 300°C. Helium was the carrier gas. Peak area of each sample was determined using the HP 3365 series II ChemStation software.

The perceptual (%) depletion of each oil component from the biotic and abiotic systems was calculated according to the following equation: 100 - (Y1.Y0^-1^) × (Z1.Z0^-1^)^-1 ^× 100, where:

- Y1 represents the surface under the chromatographic peak of samples of the inoculated cultures after 5, 10, 15 or 20 days.

- Y0 the surface under the peak of the internal standard at the same sampling time

- Z1 the surface under the chromatographic peak of sample of the inoculated or uninoculated culture at time 0.

- Z0 the surface under the peak of the internal standard at time 0 days.

The level of biodegradation of each oil component was calculated by subtracting the level of depletion in the uninoculated culture from the level of depletion in the inoculated culture. All results were presented as the mean values of three replicates from each sampling time.

### EM analysis of *P. aeruginosa *ATCC 55925

The cell-substrate physical interaction of *P. aeruginosa *ATCC 55925 growing on heating oil or pure alkanes was examined by scanning and transmission microscopy.

In the case of the scanning microscopy, samples were filtered through 0.2 μm-pore-size acetone-resistant membranes (Millipore), fixed with 5% glutaraldehyde in 0.05 M cacodylate buffer (pH 7.2) for 60 min at room temperature and dehydrated in a graded series of ethanol and acetone. Finally, samples were dried to the critical point with liquid carbon dioxide, mounted on aluminum stubs and sputter-coated with gold for analysis in a scanning microscope (digital scanning microscope Zeiss DSM 950). For the transmission microscopy study, samples were embedded in 3% (wt/v) agar, cut into 1 mm agar blocks, fixed with 3% glutaraldehyde in cacodylate buffer for 3 h and post-fixed with 1% OsO_4 _for 2 h. Samples were then dehydrated in acetone, embedded in Spurr's resin and sectioned with a diamond knife microtome (Reichert-Jung TM60). Finally, samples were stained with uranyl acetate and lead citrate for observation in a Zeis EM-10 C transmission electron microscope.

### Statistical analysis

Student's *t *test was used for statistical analysis. Samples with *P *values < 0.05 were considered statistically different.

## Results

### Heating oil profile

**Figure 1:**
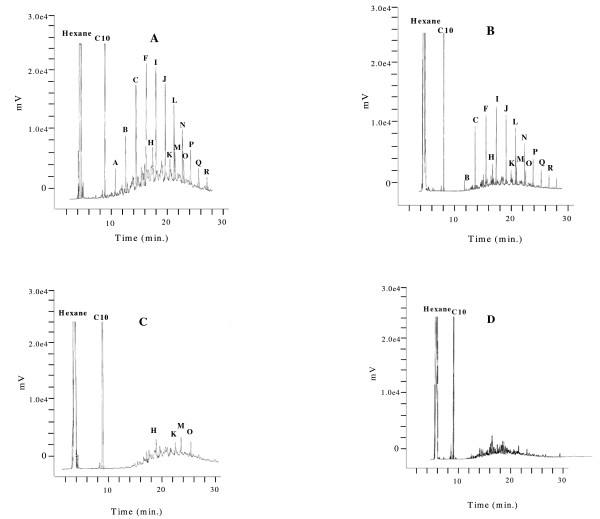
**GC profiles of saturated hydrocarbons in heating oil over time**. (A), Abiotic system without biosurfactant at T = 0 days (B), Abiotic system without biosurfactant at T = 20 days (C), Biotic system without biosurfactant at T = 20 days (D), Biotic system with biosurfactant at T = 20 days.

Gas chromatography analysis of heating oil showed a typical profile of saturated compounds. Main families of n-alkane within the profile were characterized in relation to the number of carbon atoms using a series of commercial n-alkanes from C7 to C22 carbons atoms. According to this procedure, we identified 11 n-alkanes and 4 iso-alkanes as follows: n-alkanes C11 (A), C12 (B), C13 (C), C14 (F), C15 (I), C16 (J), C17 (L), C18 (N), C19 (P), C20 (Q) and C21 (R) and iso-alkanes H, K, M and O (Figure 1A). Due to these results, we decided to use pure C10 n-alkane as the internal standard in GC since this hydrocarbon eluted just before C11 hydrocarbon and allowed easy recognition. In order to illustrate the hydrocarbon composition of the heating oil, GC profile from the abiotic system without biosurfactant is shown in Figure (1A and 1B). For the cell-free abiotic systems, all hydrocarbons showed some degree of depletion at 20 days. Particularly, hydrocarbon A (C11), the smallest n-alkane of heating oil, was nearly exhausted (Figure 1B). Abiotic depletion was taken into account to correct against the hydrocarbon loss calculated in the biotic systems and the new values were expressed as demonstrable degradation (Table 1).

**Table 1:** Loss of hydrocarbons from a heating oil in the presence of *P.aeruginosa *ATCC 55925 without and with biosurfactant at different times

H.C.*^a ^*	Demonstrable degradation of hydrocarbon without biosurfactant^c ^(%)		Demonstrable degradation of hydrocarbon with biosurfactant^c ^(%)	
**Day**	**5**	**20**	**5**	**20**

A (C11)	0 ± 0	6 ± 1	10 ± 0	49 ± 2
B (C12)	7 ± 1	34 ± 3	31 ± 2	73 ± 3
C (C13)	25 ± 1	50 ± 5	59 ± 2	85 ± 1
F (C14)	29 ± 2	73 ± 4	80 ± 5	99 ± 0
H^b^	3 ± 0	14 ± 1	60 ± 3	100 ± 0
I (C15)	23 ± 2	85 ± 3	80 ± 3	99 ± 1
J (C16)	38 ± 0	79 ± 2	77 ± 3	99 ± 1
K^b ^	13 ± 2	19 ± 0	57 ± 3	100 ± 0
L(C17)	39 ± 3	97 ± 4	79 ± 2	99 ± 0
M^b^	6 ± 0	31 ± 2	53 ± 2	100 ± 0
N(C18)	29 ± 4	97 ± 6	75 ± 6	99 ± 1
O^b^	5 ± 0	18 ± 2	60 ± 3	100 ± 0
P(C19)	28 ± 1	100 ± 0	74 ± 6	100 ± 0
Q(C20)	24 ± 6	100 ± 0	67 ± 5	100 ± 0
R(C21)	30 ± 2	100 ± 0	74 ± 4	100 ± 0

^a^Hydrocarbon. ^b^Iso-alkanes. ^c^corrected values against the abiotic loss.

### Degradation of hydrocarbons in heating oil without biosurfactant

When heating oil was exposed to *P. aeruginosa *ATCC 55925, appreciable hydrocarbon degradation was observed for some hydrocarbons, whereas others were even undetectable after 20 days of incubation (Figure 1C-D). As shown in Table 1 the biotic system without biosurfactant depicted a wide range of demonstrable n-alkanes degradation (6-100%), whereas iso-alkanes showed some degree of recalcitrance in relation to linear hydrocarbons (14-31%).

### Degradation of hydrocarbons in heating oil with biosurfactant

On the other hand, a different pattern of degradation and degradation rate were observed in the biotic system with biosurfactant (Figure 1D). It was observed that the average degradation increased from 60% without biosurfactant to 93% with biosurfactant (Table 1). The demonstrable degradation of each n-alkane increased significantly in relation to the biosurfactant-free condition (*p *< 0.05). Alkane A (C11), for which a very poor degradation was demonstrated in the biosurfactant-free condition, and iso-alkanes, which also showed to be relatively recalcitrant in the same condition, depicted from appreciable to complete hydrocarbon loss. It was clearly shown that biosurfactant-mediated dispersion enhanced degradation. With biosurfactant, partial degradation was also observed for hydrocarbons B (C12) and C (C13), whereas the other hydrocarbons (E through R) degraded completely (99-100%). In comparison with the biotic systems with biosurfactant, non-dispersed cultures showed partial degradation for alkanes A through O and only higher molecular weight alkanes P through R were completely exhausted.

### Degradation of individual hydrocarbons with and without biosurfactant

In order to access the patters of degradation of the same kind of hydrocarbon species under different conditions, C7-C19 alkanes were added individually to the degradation systems so that each hydrocarbon became the sole carbon source. As shown in Table 2 hydrocarbon degradation was only demonstrated from C12 to C19 hydrocarbons, for which average depletion significantly increased from 24% without biosurfactant to 53% with biosurfactant (*p *< 0.05). No degradation could be proved with hydrocarbons C7-C11 regardless of the presence of biosurfactant, as they depleted completely in the abiotic systems. In terms of the overall profile of degradation, no notorious difference was observed in relation to hydrocarbon in the heating oil. It was interesting that hydrocarbon C19 (pristane) did not degrade without biosurfactant, but did so in its presence. In contrast to C7 through C11 hydrocarbons, for which degradation could not be demonstrated due to complete depletion in the abiotic systems, pristane was completely degraded (100%) when dispersed into the aqueous phase.

**Table 2:** Loss of individual hydrocarbons (C7-C19) in the presence of *P. aeruginosa *ATCC 55925 without and with biosurfactant at different times

H.C.*^a ^*	Demonstrable degradation of hydrocarbon without biosurfactant^c ^(%)		Demonstrable degradation of hydrocarbon with biosurfactant^c ^(%)	
**Day**	**5**	**20**	**5**	**20**

C7	ND^e^	ND	ND	ND
C8	ND	ND	ND	ND
C9	ND	ND	ND	ND
C10	ND	ND	ND	ND
C11	ND	ND	ND	ND
C12	4±2	23±1	12±4	8±2
C13	8±2	64±2	57±4	92±2
C14	11±1	70±6	81±3	100±0
C15	6±0	41±3	87±5	100±0
C16	5±0	39±3	90±4	100±0
C17	7±0	51±4	89±3	100±0
C19^b^	0± 0	0±0	65±5	100±0

^a ^Hydrocarbon. ^b ^Branched alkane (pristane). ^c ^Corrected against the abiotic hydrocarbon loss. ND: Not determined due to high abiotic depletion.

### Degradation of hydrocarbons in an artificial mixture with and without biosurfactant

The same alkanes C7 through C19 were combined in an artificial mixture to partially mimic heating oil, though lower molecular weight alkanes were also included (C7-C10). A different pattern of hydrocarbon loss was observed in relation to the same n-alkanes added individually (Table 3). For instance, among C12-C17 hydrocarbons, degradation tended to decrease as the molecular weight increased, whereas the same hydrocarbon species in the artificial mixture degraded the other way around, that is, degradation increased as hydrocarbons became of bigger molecular weight. As stated above for individual hydrocarbons, the degradation of C7-C11 compounds from the mixture could not be demonstrated even in the presence of biosurfactant. In all cases there was a significant enhancement of demonstrable degradation when hydrocarbons were dispersed into the aqueous phase (*p *< 0.05). Under this condition, it was shown that the mean degradation value increased from 39% without biosurfactant to 50% with biosurfactant. It is worth noting that in contrast to the recalcitrance of C19 hydrocarbon as the sole carbon source without biosurfactant, this multi-branched alkane was degraded in the artificial mixture regardless of the tensio-active agent. It thus appeared that the loss of this hydrocarbon was enhanced by other ready-usable hydrocarbons in the mixture (n-alkanes). For middle and high molecular weight hydrocarbons no correlation was found between the percentage of degradation and hydrocarbon chain length. However, the biosurfactant always increased total hydrocarbon loss as well as the overall rate of degradation as seen by shorter times of removal under different conditions.

**Table 3:** Loss of hydrocarbons from an artificial mixture (C7-C19) in presence of *P. aeruginosa *ATCC 55925 without and with biosurfactant at different times

H.C.*^a^*	Demonstrable degradation of hydrocarbon without bio biosurfactant^b ^(%)		Demonstrable degradation of hydrocarbon with biosurfactant^b ^(%)	
**Day**	**5**	**20**	**5**	**20**

C7	ND^e^	ND	ND	ND
C8	ND	ND	ND	ND
C9	ND	ND	ND	ND
C10	ND	ND	ND	ND
C11	ND	ND	ND	ND
C12	0 ± 0	52 ± 7	0 ± 0	84 ± 4
C13	7 ± 1	42 ± 8	26 ± 3	93 ± 4
C14	17 ± 2	65 ± 4	60 ± 4	98 ± 9
C15	13 ± 2	67 ± 2	87 ± 3	96 ± 2
C16	17 ± 3	74 ± 6	95 ± 8	96 ± 4
C17	21 ± 3	74 ± 9	88 ± 7	93 ± 5
C19^c^	0 ± 0	49 ± 2	81 ± 8	88 ± 3

^a ^Hydrocarbon. ^b ^Corrected against the abiotic hydrocarbon loss. ^c ^Branched alkane (pristane). ND: Not determined due to high abiotic depletion.

### EM analyses (SEM and TEM) of *P. aeruginosa *ATCC 55925 growing on hydrocarbons

**Figure 2:**
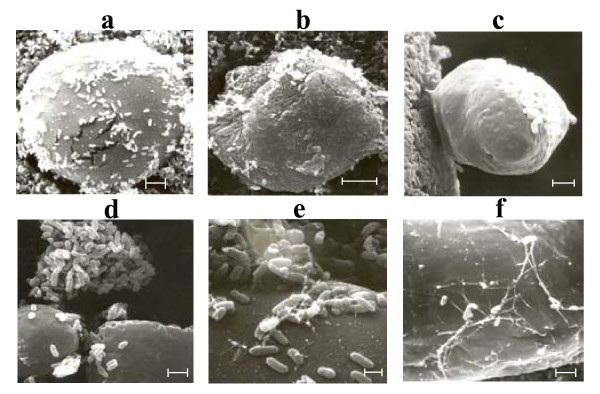
**SEM study of *P. aeruginosa *ATCC 55925 adhering to polymeric spheres covering oil-in water emulsion droplets while growing on hydrocarbons**. Bacteria are seen adhered on the spheres surface (a), embedded in several polymeric layers (b), projecting out from a cell cluster on the sphere surface (c) linked as cell clusters (d) and by appendages (d and f). Scale bars: a-b 5 μm, c-e 2 μm, f 1 μm.

SEM analysis permitted a tri-dimensional observation of *P. aeruginosa *ATCC 55925 growth on alkanes. This bacterial strain showed different adaptable responses to hydrocarbon growth, and was found either free within the bulk water phase or associated with hydrocarbons in the oil/water interface and emulsions (Figure 2a-f). Cells were seen densely gathered around polymeric spheres of bacterial origin (Figure 2a), embedded in several polymeric layers below the sphere surface (Figure 2b), on the surface of spheres projecting out from a cell cluster or biofilm (Figure 2c), linked together as clusters (Figure 2d), or by long extracellular appendages (Figure 2e and 2f), and individually adhered by extracellular appendages over the sphere surface (Figure 2a, d, e and 2f). Even though the outermost layer of the spheres appeared smooth, lower cell layers, which were revealed as the electron bean passed through the samples, had a rough appearance (Figure 2b). Theses structures were not seen in cultures of *P. aeruginosa *ATCC 55925 growing on non-hydrocarbon substrates (not shown) and particularly developed around the emulsified oil droplets.

**Figure 3:**
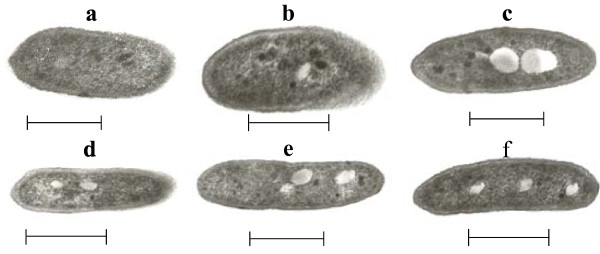
TEM of *P. aeruginosa* ATCC 55925 showing different patterns of inclusions in relation to control: growing on PYG culture medium (a), growing on diesel oil (b-d) and C13 hydrocarbon (e-f). Inclusions are only depicted in several samples of *P. aeruginosa* ATCC 55925 growing in oil-based culture medium (b-f). Absence of such inclusions is noted in cultures grown in rich PYG medium without oil (a). Scale bars: 1µm.

TEM study revealed the appearance of non-membrane-bounded cytoplasmatic electron-transparent inclusions (Figure 3b-f), which were absent in glucose based-cell cultures (Figure 3a). These large spherical structures were similar to those reported previously for *Rhodococcus opacus, Acinetobacter calcoaceticus *and *Mycobaterium *(Alvarez et al. 1996; Marin et al. 1996, Solano-Serena et al. 2000, respectively), which indicated that the formation of this type of inclusions may be a general cell adaptation to hydrocarbon growth. Light microscopy revealed that the cell growth was more concentrated around the oil droplets than in the water phase (data not shown), which indicated that *P. aeruginosa *ATCC 55925 was chemotactically attracted towards the alkanes (Baldi et al. 1999).

## Discussion

*Pseudomonas aeruginosa *ATCC 55925 was used in this study because of its ability to produce a potent tensio-active agent in a rich culture medium and because of its potential to biodegrade a wide variety of hydrocarbon compounds (Rocha and Infante 1997; Rocha et al. 1999; Rocha et al. 2000).

Hydrocarbon degradation has been usually reported as total saturate or aromatic loss. In this study we determined the fate of each hydrocarbon under different conditions in other to establish a pattern of biodegradation. *P. aeruginosa *ATCC 55925 showed different patterns of alkane biodegradation in the context of a single aliphatic compound present alone or as part of different hydrocarbon mixtures, such as a heating oil and an artificial mixture of alkanes. Also, the alkane chain length, alkane branching and the biosurfactant-mediated dispersion of alkanes into the aqueous medium were investigated.

Since no low molecular weight-hydrocarbon species were found in the heating oil (which has been reported to be either toxic to cells or volatile) all n-alkane species degraded and supported cell growth, while iso-alkanes showed some degree of recalcitrance. In this latter case, methylation of alkanes, as in iso-alkanes, could have decreased the solubility of the aliphatic compounds, which in turns would have rendered resistance to or discouraged biodegradation. This is especially true when methylation occurs at the saturate β-carbon, which is known to inhibit β-oxidation unless the bacterial population is able to β-descarboxymethylate (Schaeffer et al. 1979; Singer and Finnerty 1984; Berekaa and Steinbüchel. 2000). In addition, n-alkanes probably inhibited iso-alkane degradation as previously reported (Marin et al. 1995). However, we also showed in this study that n-alkanes could instead enhance the biodegradation of branched alkanes.

On the other hand, the use of the biosurfactant significantly enhanced degradation of all alkane species, including recalcitrant iso-alkanes. These results suggested that biosurfactant-mediated dispersion of hydrocarbons played a very important role in the degradation of saturated compounds (Neu 1996; Bruheim and Eimhjellen 2000; Noordman and Janssen 2002), regardless of the metabolic strategy used by the bacterial population. In the case of iso-alkanes, biosurfactant-induced emulsions probably compensated the reduction of hydrocarbon solubility caused by methyl branching, which would have lowered substrate availability to cells. Highly volatile alkanes showed the highest hydrocarbon loss in the abiotic systems, and hence, the lowest demonstrable degradation in the biotic systems. Contrary to what we expected, the biosurfactant did not seem to affect volatilization of low molecular weight hydrocarbons. According to these results we suggest that the biosurfactant increased the low solubility caused either by methyl branching or by the carboxylic derivative obtained at the initial oxidation steps of alkanes when they became slow-moving compounds.

Pristane, a low solubility multi-methyl branched alkane, usually remains recalcitrant in biodegradation systems, and it is even used as an internal marker to determine biotic hydrocarbon loss. In this study, the recalcitrance of pristane observed under some conditions suggested that low solubility and probably the substitution pattern after several cycles of β-oxidation would have inhibited oxidation. Particularly, the methyl substitutions at carbon 3 would have rendered pristane recalcitrant, unless they were bypassed by a β-decarboxymethylation event (Cantwell et al. 1978). Opposed to those results, our data suggested that the biosurfactant and the presence of some types of n-alkanes directly enhanced degradation of pristane by increasing its solubility and indirectly by allowing pristane to reach more easily the β-oxidation steps. This novel result contrasted with previous reports which indicated that n-alkanes inhibited the biodegradation of methyl branched alkanes (Leahy and Colwell 1990). It was therefore shown that in terms of net degradation value and pattern of degradation, alkanes behaved differently depending on whether they were a unique carbon source or part of a particular hydrocarbon mixture (natural or artificial), indicating that several types of hydrocarbon-hydrocarbon and hydrocarbon-cell interactions occurred.

In addition, it was demonstrated in this study that the different patterns of biodegradation became similar when hydrocarbons were dispersed by the biosurfactant. Even though it has been reported that biosurfactants usually enhance biodegradation of single hydrocarbons, it is also known that micellar solubilization can affect the biodegradation of hydrocarbon mixtures depending upon their ability to partition into the micellar core. In mixed systems, alkanes compete among themselves to partition into the micelle and a decreased rate of degradation may result due to exclusion, or very low levels of solubilization within the micelle (Kniemeyer et al. 2003). By the contrary, our results demonstrated that biosurfactant enhanced biodegradation of alkanes under all conditions.

Since no detectable bacterial growth was associated with low molecular weight alkanes in any biotic system (data not shown) and considering that such hydrocarbons were exhausted in the abiotic systems due to volatilization, we suggest that substrate unavailability was the main limiting factor that affected the time-course and fate of such hydrocarbons. Nevertheless, the toxicity of low molecular weight alkanes (Solano-Serena et al. 2000) or the lack of capability of *P. aeruginosa *to degrade these hydrocarbons (Scott and Finnerty 1976) cannot be ruled out with the data at hand.

These findings report neatly the different patterns of biodegradation and the fate of particular n-alkanes when they impact individually or as part of an alkane mixture, together with the effect of a biosurfactant under such conditions. These results would impact the expectations and interpretation of the alkane degradation under the context of bioremediation.

It was also shown in this study that *P. aeruginosa *ATCC 55925 depicted interesting cell strategies to degrade hydrocarbons, such as biosurfactant (Marin et al. 1995; Wolfaardt et al. 1998) and non biosurfactant-mediated cell surface changes as well as the formation of inclusions. SEM analysis revealed several types of extracellular bacterial structures when *P. aeruginosa *ATCC 55925 was grown on hydrocarbons, probably to increase the substrate surface area, and hence, to facilitate biodegradation. Based on our results we propose for the first time, to our best knowledge, that cell clusters and cell flocks were part of an initial phase in the formation of the final spherical structures surrounding the oily substrate. Wu and Ju (1997) and Whyte et al. (1998) have reported this type of cell clusters and cell flocks as unique cell adaptations while growing on hydrocarbons, suggesting a cross-linked polymeric structure. However, these authors failed to demonstrate the step-by-step formation of the final polymeric spheres. Based on our evidence, we have suggested that cells linked to each other by extracellular appendages could participate in the waxy particles formation and eventually in the formation of the final polymeric spheres through the addition of successive cell layers. All these results suggest that *P. aeruginosa *ATCC 55925 exhibited many structural changes at the cell surface level, some of them probably mediated by the biosurfactant, and certainly in combination with biosurfactant as strategies to adapt to oily substrates. It is worth noting that the production of extracelullar polymeric substances, changes in the fatty acid composition of membranes (Garnier et al. 2000) and cell appendages (Scott and Finnerty 1976; Kretschmer and Wagner 1982; Takeda et al. 1991; Whyte et al. 1999; Wolfaardt et al. 1994; Marin et al. 1996) have been reported previously as responses of bacterial growth on hydrocarbons. However, the multi-layer composition of the polymeric spheres observed in the study is, to our best knowledge, the first report of such structural pattern. Even though cell hydrophobicity was not investigated in this study, it has been reported to occur on *P. aeruginosa *strains growing on hydrocarbons by altering the LPS or shortening LPS O-antigen on the cell surface (Zhang and Miller 1994; Al-tahhan et al. 2000; Norman et al. 2002).

TEM observation also revealed intracellular transparent vesicles not depicted in cell cultures growing on non-hydrocarbon culture media. We were unable, with the data at hand, to determine the nature of these inclusions and their content. Nevertheless, it has been speculated that these structures could contain metabolic waste from the hydrocarbon catabolism or may function as reservoirs for untouched hydrocarbons (Marin and Laborda 1996). Other energy-dependent mechanisms (efflux-influx) and metabolic strategies such as reduction of low molecular weight aliphatic toxicity could also be involved in *P. aeruginosa *cultures growing on alkanes. None of these could be ruled out in this study.

In summary, different patterns of hydrocarbon degradation and cell strategies were shown by *P. aeruginosa *ATCC 55925 growing on aliphatic compounds as the sole carbon and energy source. Susceptibility of alkanes to degradation depended upon the presence of other readily available hydrocarbons, type of hydrocarbon, dispersion into aqueous phase, hydrocarbon volatilization, cell metabolic pathways and several structural changes from inclusions to complex extracellular polymeric spheres. This study investigated total hydrocarbon loss as well as individual alkane utilization in terms of specific patterns of microbial and biosurfactant mediated-biodegradation and cell adaptations to hydrocarbon growth. We believe that determining different degradation profiles for specific hydrocarbon families under different conditions will contribute to improving oil bioremediation techniques.

## Competing interests

The authors declare that they have no competing interests.

## Authors' contributions

CA conceived the study, carried out the design and the execution of the biodegradation experiments and executed the electron microscopy studies. AP participated in the design of the electron microscopy studies. FL participated in the design and coordination of the study. All authors read and approved the final manuscript.
